# Vascular Remodeling During Late-Gestation Pregnancy: An In-Vitro Assessment of the Murine Ascending Thoracic Aorta

**DOI:** 10.1115/1.4064744

**Published:** 2024-07-01

**Authors:** Ana I. Vargas, Samar A. Tarraf, Turner Jennings, Chiara Bellini, Rouzbeh Amini

**Affiliations:** Department of Bioengineering, Northeastern University, Boston, MA 02115; Department of Bioengineering, Northeastern University, Boston, MA 02115; Department of Mechanical and Industrial Engineering, Department of Bioengineering, Northeastern University, Boston, MA 02115; Department of Bioengineering, Northeastern University, Boston, MA 02115; Department of Mechanical and Industrial Engineering, Department of Bioengineering Northeastern University, Boston, MA 02115

## Abstract

Maternal mortality due to cardiovascular disease is a rising concern in the U.S. Pregnancy triggers changes in the circulatory system, potentially influencing the structure of the central vasculature. Evidence suggests a link between a woman’s pregnancy history and future cardiovascular health, but our understanding remains limited. To fill this gap, we examined the passive mechanics of the murine ascending thoracic aorta during late gestation. By performing biaxial mechanical testing on the ascending aorta, we were able to characterize the mechanical properties of both control and late-gestation tissues. By examining mechanical, structural, and geometric properties, we confirmed that remodeling of the aortic wall occurred. Morphological and mechanical properties of the tissue indicated an outward expansion of the tissue, as reflected in changes in wall thickness (~ 12% increase) and luminal diameter (~ 6% increase) at its physiologically loaded state in the pregnant group. With these geometric adaptations and despite increased hemodynamic loads, pregnancy did not induce significant changes in the tensile wall stress at the similar physiological pressure levels of the pregnant and control tissues. The alterations also included reduced intrinsic stiffness in the circumferential direction (~ *18*%) and reduced structural stiffness (~ 26%) in the pregnant group. The observed vascular remodeling maintained the elastic stored energy of the aortic wall under systolic loads, indicating preservation of vascular function. Data from our study of pregnancy-related vascular remodeling will provide valuable insights for future investigations of maternal cardiovascular health.

## 1 Introduction

The intersection of pregnancy and cardiovascular disease (CVD) is a critical area of study, as CVD poses a major risk during pregnancy, affecting a substantial proportion of expectant mothers worldwide. In the U.S., CVD is responsible for more than a third of maternal mortality instances [1–3]. The alarming statistics of maternal mortality due to CVD underline the urgency of addressing cardiovascular health during pregnancy as a mean to improve overall maternal care. In addition, maternal cardiovascular health is intricately connected to fetal well-being. The maternal cardiovascular system delivers oxygen and nutrients to the developing fetus. Any compromise in this system due to CVD can impact fetal growth and development, potentially leading to adverse outcomes for the baby [4–6]. As such, addressing CVD during pregnancy is vital not only for the well-being of the mother but also for the future health of the child.

To identify the underlying causes and risk factors associated with CVD in pregnancy, it is essential to conduct a comprehensive assessment of the physiological changes that occur in a healthy, uncomplicated pregnancy. Interestingly, pregnancy induces significant hemodynamic changes that may lead to cardiovascular system remodeling. In normal pregnancies, the following maternal hemodynamic observations have been made during gestation:

- Blood pressure does not change significantly and pulse pressure also remains constant [7–9].
- Cardiac output increases by approximately 45% [7,10–13].
- There is an approximate increase of 40% in the total blood volume by the end of gestation [14,15].
- At midgestation, the blood becomes less viscous. The viscosity, however, reverts to normal levels during late gestation, accompanied by a 34% reduction in peripheral resistance [7,11,13,16–18].

**Fig. 1:**
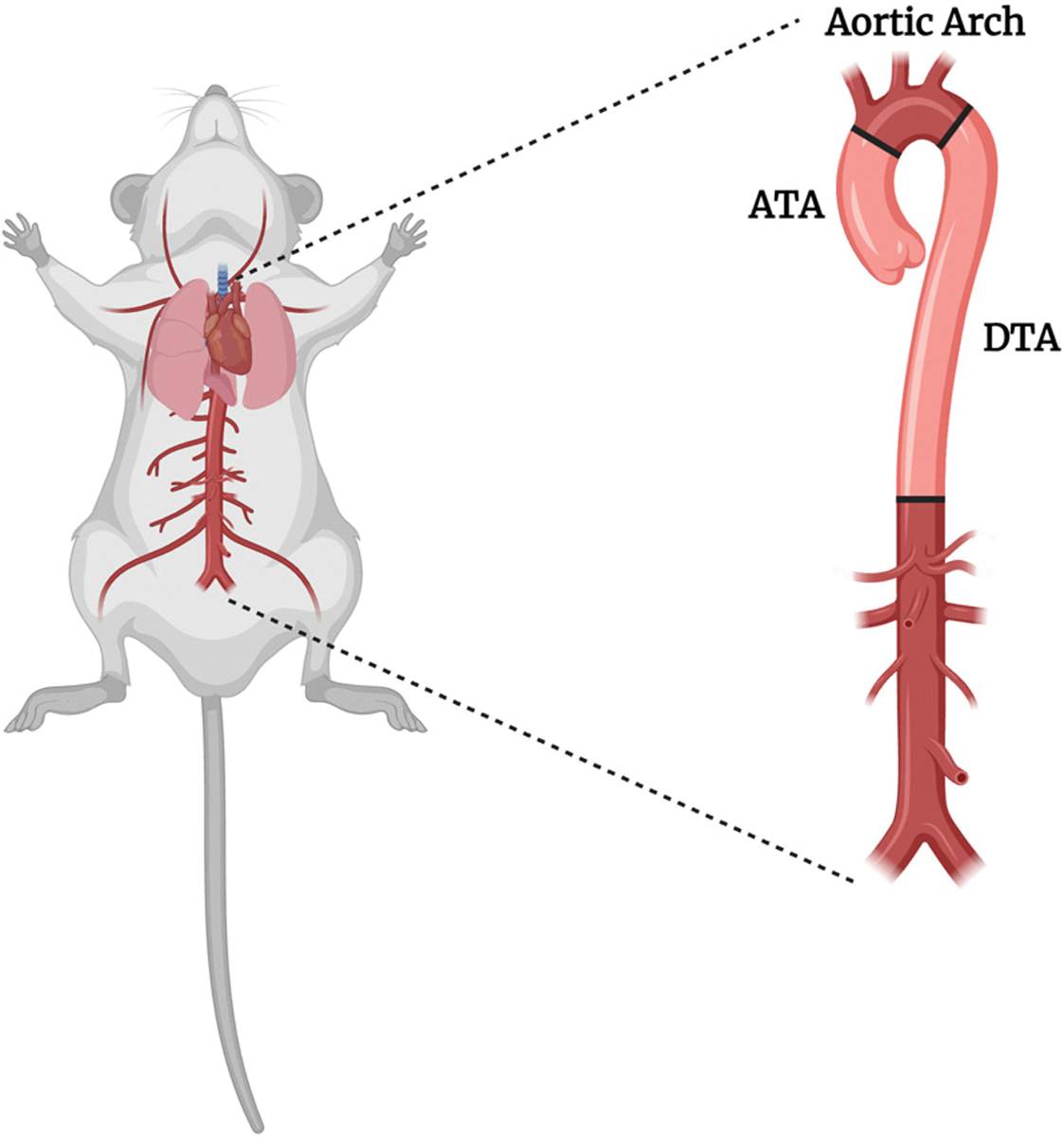
Schematic view of the thoracic aorta in the mouse. The ascending thoracic aorta (ATA) begins at the aortic valve, originating from the left ventricle of the heart, and it ascends in the chest, supplying oxygenated blood to the coronary arteries. The descending thoracic aorta (DTA) continues from the aortic arch, coursing downward along the spine in the chest cavity, supplying oxygenated blood to the thoracic structures. The image was made in Bio-Render.com.

Structural adaptations in maternal arteries are necessary to accommodate altered flow and volume [19]. In the realm of vascular mechanobiology, structural adaptation due to hemodynamic changes and altered flow is a well-known phenomenon. For example, it is known that hypertension leads to the restructuring of collagen within the extracellular matrix [20–23]. While the precise connection between arterial stiffening and hypertension remains a topic of ongoing discussion [24], one argument is that elevated mechanical stress on vascular tissues resulting from hypertension could be the reason behind vascular structural modifications. Similarly, alterations in blood flow can trigger changes in the luminal diameter of the vessel to maintain homeostatic shear stress within the aortic wall [25]. This flow-induced adaptation encompasses diverse mechanisms, mostly characterized by the growth, remodeling, or degradation of the vessel wall in the circumferential direction [26–28]. When it comes to pregnancy, however, our knowledge of vascular adaptation is scarce. Accurate quantification of aortic wall stiffness is essential to bridge knowledge gaps regarding vascular remodeling in normal pregnancies. Such quantification will also assist in identifying the etiology of adverse events (e.g., aortic aneurysm) in hypertensive pregnancies or during pregnancies in individuals with other underlying diseases such as connective tissue disorders. Clinically, aortic compliance and stiffness during pregnancy have been studied using pulse wave velocity. Structural stiffness is a measurement of vessel wall elastic responses, and it is influenced by changes in the geometry, intrinsic stiffness, and loads applied on the vessel wall. Intrinsic stiffness, on the other hand, is an inherent property of the arterial wall that is primarily determined by the composition and organization of its structural components. In our recent work [29], we have shown—for the first time—that the murine descending thoracic aorta (DTA) undergoes remodeling characterized by an increase in luminal diameter, wall thickness, and tissue softening in late gestation. The ascending thoracic aorta (ATA), which is adjacent to the heart (Fig. 1), also undergoes significant changes following pregnancy [19,30]. In other examples of vascular remodeling, it has been shown that central arteries that are closer to the heart are influenced more by the alterations in the cardiac function as compared to those that are further down the stream [31]. As such, we hypothesized to observe significant changes in ATA geometry, structural stiffness, and intrinsic stiffness in late gestation.

## 2 Materials and Methods

### 2.1 Animal and Sample Groups.

All procedures involving animals were conducted in compliance with the National Institutes of Health regulations and were approved by the Northeastern University Institutional Animal Care and Use Committee (IACUC). A power analysis was conducted using G*Power version 3.1.9.6 [32,33] for sample size estimation based on data from our recent work [29] in which we compared late-gestation DTA samples to age-matched control DTA samples. Our analysis suggested that using n=10 mice per group would be sufficient to estimate differences in the mean of stress and distensibility with a power of 0.75 and geometric changes with a power greater than 0.95. Since we expected the changes observed in the ATA to be similar to those observed in the DTA, the obtained sample size of n=10 was deemed adequate to test the study hypothesis. For the late-gestation group, we used wild-type pregnant C57BL/6 mice at gestation day 18 ± 1 (n=10). The age-matched control group consisted of nulligravida (never-pregnant) C57BL/6 mice aged 10 ± 1 weeks (n=10). It is well-known that various wild-type mice may exhibit distinct mechanical properties in their vascular tissues [34,35]. Consequently, in the absence of genetic mutations, multiple options of wild-type mice are available for studying the mechanobiological remodeling of the aorta during pregnancy. We specifically selected wild-type C57BL/6 because our recently published work [29] has confirmed the suitability of these mice as an appropriate model for investigating vascular remodeling during pregnancy.

### 2.2 Hemodynamics Measurements.

For each mouse, blood pressure was measured noninvasively using a tail cuff system (CODA; Kent Scientific, Torrington, CT) without anesthesia, as described previously [29,36]. The pressure measurement approach necessitated a gentle form of restraint, achieved by positioning the mouse within a see-through plastic cylinder with its tail exposed at one end. To acclimate the mice and minimize the impact of emotional distress on the measurements, mice were placed on the restraints for 20 min per day, for 3 days. Following the training, actual measurements were taken, with each session typically lasting between 15 and 30 min, depending on the level of excitability of the mouse. The pulse pressure (ΔP) was subsequently calculated for each animal

(1) $$\Delta P=P_{sys}-P_{dias}$$

where $P_{\text{sys}}$ and $P_{\text{dias}}$ were systolic and diastolic blood pressures, respectively.

### 2.3 Sample Preparation.

**Fig. 2:**
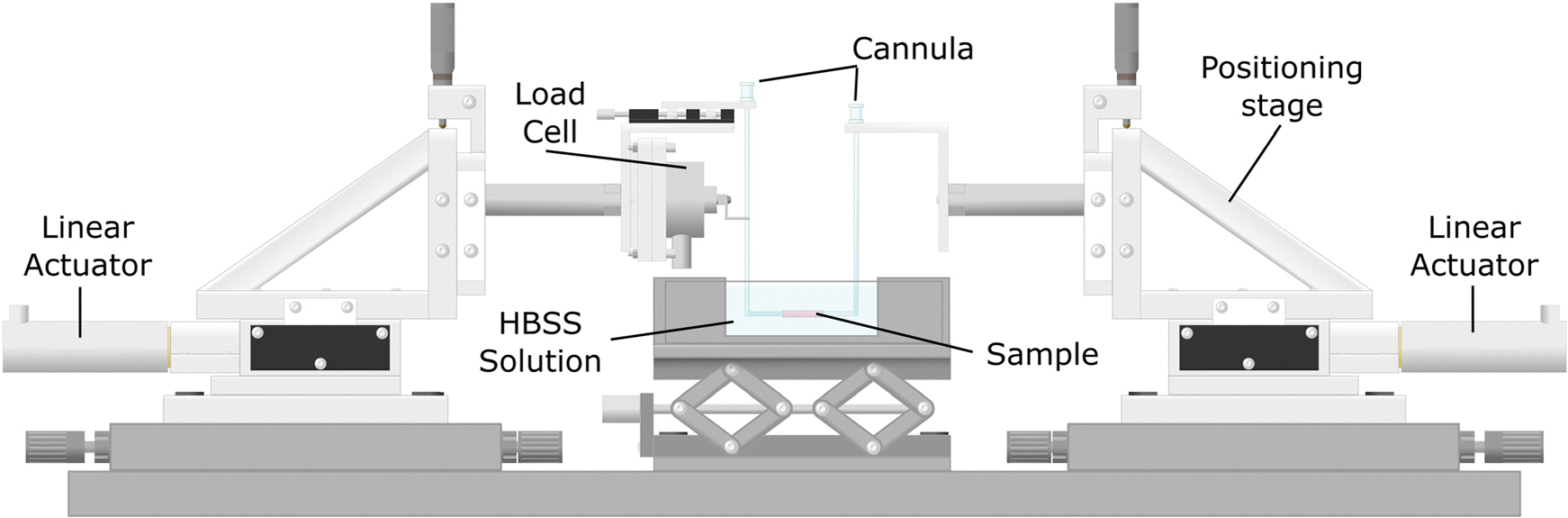
Schematic of the biaxial testing device used for inflation–extension testing. The tissue sample is securely positioned within a bath filled with Hank’s buffer solution, with glass cannulas affixed to both ends. Linear actuators, seen at each end of the device, are linked to the positioning stage and allow the axial stretching of the sample. A load cell attached to the distal end cannula by a hook and secured with paraffin wax is used to measure the force resistance of the stretching tissue.

Following blood pressure collection, the animals were euthanized. The ATAs were then carefully excised using a dissection microscope. To prepare the tissue for testing, a number of vascular segments had to be tied using 9–0 nylon suture. These segments included the proximal section of the DTA, the left common carotid artery, and the left subclavian artery. Prepared tissue samples were cannulated into glass micropipettes and affixed to a custom-made mechanical testing device (Fig. 2) controlled by an internally developed computer program [29,37–39].

### 2.4 Biaxial Testing.

Mechanical testing of the tissues was performed on a biaxial pressure-extension system shown in Fig. 2. Cannulated samples were placed in a bath of Hank’s Balanced Salt Solution (HBSS). A camera was setup in front of the clear bath to gauge the outer diameter of the specimen. Linear actuators attached to the stage of the device were used to control the distance between the two cannulas and, as a result, the stretching of the tissue along its axis. To assess force resistance to tissue stretching, a load cell (in series with a wire hook) was affixed to the cannula and firmly held in place by applying paraffin wax. The luminal pressure was controlled by a hydraulic system with two transducers in line. A custom computer interface generated using LabVIEW (National Instruments, Austin, TX) was employed to control the pressure and stretch applied on the tissue and to simultaneously collect data on diameter and force.

To estimate the in vivo axial stretch of each sample, we identified the particular stretching level where the applied force did not change when the pressure ranged from 80 to 120 mmHg (physiological pressures) [40]. After determining this in vivo axial stretch, the tissue samples were subjected to an acclimation and preconditioning protocol, as described previously [29].

The tissue samples were then subjected to two types of passive testing—pressure-diameter (p-d) and force-length (f-l) tests. P-d tests consisted of cyclic pressure changes, i.e., ranging between a minimum value of 10 mmHg and a maximum value of 140 mmHg, at three specific axial stretch levels: (i) the estimated in vivo axial stretch, (ii) 5% above the in vivo axial stretch value, and (iii) 5% below the in vivo axial stretch value. F-l tests were performed by maintaining the luminal pressure at either 10, 60, 100, or 140 mmHg while stretching the tissue sample to obtain a specific axial force value (from −0.5 g up to 5.50 g). During all of these testing protocols, parameters representing mechanical loading (i.e., the luminal pressure and axial force) and those representing deformation (i.e., the outer diameter and axial stretch) were continuously measured and recorded in real-time. While selecting loading protocols tailored to the individually measured blood pressure of each animal may be a physiologically relevant approach, such a method requires multiple labor-intensive blood pressure measurements that are not feasible within the experimental constraints. More importantly, in the mechanical testing of biological tissues, the preference often lies in employing consistent loading protocols informed by averaged values of in vivo physiological loads, as opposed to animal-specific ones. Such an approach facilitates meaningful comparisons across various studies conducted at different times [41–44]. After the tests were completed, a ring sample was sliced and imaged to measure the unloaded wall thickness.

### 2.5 Biomechanical Analyses.

In accordance with the procedures outlined in our previously published works [29,39], we assessed the mechanical characteristics of the aortic wall by analyzing the stress–stretch behavior of the tissue. We calculated the circumferential stretch ($\lambda_{\theta }$) and axial stretch ($\lambda_{z}$) for each individual sample, using the deformed state of the tissue and its corresponding original, traction-free (free from external loads and prestress) state

(2) $$\lambda_{\theta }=\frac{r\;+\;\frac{h}{2}}{R\;+\;\frac{H}{2}}\;\text{and}\;\lambda_{z}=\frac{l}{L}$$

where r,h, and l were the radius of the inner luminal portion, the thickness of the tissue wall, and the length along the axis of the tissue when it was in the loaded deformed configuration, respectively. Correspondingly, R,H, and L represented the same measurements of the same sample in their original, traction-free state. The average volume of the tissue was calculated in the traction-free geometry where outer diameter, axial length, and wall thickness were measured. The determined volume was subsequently employed to calculate the wall thickness of the vessel in its loaded configuration, wherein the incompressibility constraint was applied. In other words, we made the assumption that the tissue volume remained constant during the transition from the traction-free state to the loaded configuration.

The circumferential ($\sigma_{\theta \theta }$) and axial stretch ($\sigma_{zz}$) components of the Cauchy stress were then calculated

(3) $$\sigma_{\theta \theta }=\frac{Pr}{h}\;\text{and}\;\;\sigma_{zz}=\frac{f}{\pi h(2r\;+\;h)}$$

with P being the experimental values of pressure and f the total axial force. The circumferential stress was computed using Laplace’s law (assuming that the aorta can be approximated as a thin-walled cylinder) [45]. We calculated the total axial force f, used in Eq. (3), as it follows:

(4) $$f=f_{T}+\pi r^{2}P$$

for which both the contribution of the pressure and that of $f_{T}$, the axial force measured from the force transducer, were accounted. The aorta was modeled as a hyperelastic material and biaxial data from individual unloading p-d and f-l tests were used to estimate the best-fit coefficients of a validated strain energy density function [46,47], in the form

(5) $$W(C)=\frac{c}{2}\left(I_{c}-3\right)+\sum_{i=1}^{4}\;\frac{c_{1}^{i}}{4c_{2}^{i}}\left\{e^{c_{2}^{i}{\left(IV_{c}^{i}-1\right)}^{2}}-1\right\}$$

where the first neo-Hookean term characterizes the isotropic portion of the tissue, represented by the coefficient c (measured in stress units). The subsequent terms, expressed in a Fung-type exponential form, take into account the influence of circumferential smooth muscle fibers and the four families of collagenous fibers ($c_{1}^{i}$’s have the unit of stress and $c_{2}^{i}$’s are dimensionless). The superscript i indicates the direction corresponding to the four-fiber family model (1 = axial, 2 = circumferential, 3, 4 = two symmetric diagonals). Elastic energy was calculated by numerically integrating the strain energy density function (Eq. (5)) over a deformed volume, using the specific deformation field associated with the loaded configuration of interest.

The strain energy density (as shown in Eq. (5)) depends on the right Cauchy–Green deformation tensor C

(6) $$C_{IJ}=F_{kI}F_{kJ}$$

with F being the deformation gradient tensor defined by

(7) $$F=\left[\begin{array}{ccc} \lambda_{r} & 0 & 0\\ 0 & \lambda_{\theta } & 0\\ 0 & 0 & \lambda_{z} \end{array}\right]$$

The radial stretch was calculated using the assumption of incompressibility (i.e., detF=1)

(8) $$\lambda_{r}=\frac{1}{\lambda_{\theta }\lambda_{z}}$$

The strain energy function made use of two invariants of the right Cauchy–Green deformation tensor C [39,46]. The first invariant was defined by

(9) $$I_{C}=C_{II}=\lambda_{\theta }^{2}+\lambda_{z}^{2}+\frac{1}{\lambda_{\theta }^{2}\lambda_{z}^{2}}$$

The fourth invariant, characterizing deformation along the specific orientation of fibers within the tissue, was defined by

(10) $$IV_{C}^{i}=\lambda_{\theta }^{2}{sin}^{2}\alpha_{0}^{i}+\lambda_{z}^{2}{cos}^{2}\alpha_{0}^{i}$$

where the angle $\alpha_{0}^{i}$ was chosen in relation to the axial orientation within the initial reference configuration. Consequently, the axial and circumferential fiber groups were oriented at angles of $\alpha_{0}=0^{\circ }$ and 90°, correspondingly.

Using the strain energy function (Eq. (5)), the Cauchy stress tensor σ was then computed

(11) $$\sigma_{ij}=p\delta_{ij}+2F_{iK}F_{jL}\frac{\partial W}{\partial C_{KL}}$$

where $\delta_{ij}$ is the kronecker delta, or the component form of the identity tensor I and p is a Lagrange multiplier, enforcing the incompressibility constraint. We employed a nonlinear regression model to minimize the error between measured and predicted values of pressure and axial force. This model enabled us to estimate the eight material parameters outlined in Eq. (5). The theoretical pressure and axial force values for a specific stress state used in this approach for data fitting and error minimization were obtained using Eqs. (3) and (4). We repeated this procedure for each collected dataset, to estimate specimen-specific parameters, as well as for the average data sets, to estimate group-specific parameters (i.e., late gestation pregnant versus age-matched control).

We used the specimen-specific best-fit material parameters to predict the mechanical variables of interest under a particular loading condition, specifically the systolic blood pressure unique to each group and the energetically-favorable axial stretch unique to each sample [34], consistent with our previous work [39,48]. In particular, the biomechanical parameters of interest include geometric parameters such as thickness and luminal diameter, structural and material metrics, such as stress, stretch, and stiffness, both in the circumferential direction and in the axial direction, and vascular function metrics such as distensibility and elastic energy.

To calculate the circumferential (${𝒞}_{\theta \theta \theta \theta }$) and axial (${𝒞}_{zzzz}$) components of the fourth-order linearized (intrinsic) stiffness tensor, we employed a theory referred to as “small-on-large,” described by Baek et al. [46]. This theory relies on linear elasticity approximations to depict how the aortic wall responds to the cardiac cycle. Using the strain energy function shown in Eq. (5), we employed the following expression to calculate the components of the fourth-order linearized stiffness tensor ${𝒞}_{ijkl}$:

(12) $${𝒞}_{ijkl}\;={\left.2\delta_{ik}F_{lA}^{o}F_{jB}^{o}\frac{\partial W}{\partial C_{AB}}\right|}_{C^{o}}\;\;+{\left.2\delta_{jk}F_{iA}^{o}F_{lB}^{o}\frac{\partial W}{\partial C_{AB}}\right|}_{C^{o}}\;\;+{\left.4F_{iA}^{o}F_{jB}^{o}F_{kP}^{o}F_{lQ}^{o}\frac{\partial^{2}W}{\partial C_{AB}\partial C_{PQ}}\right|}_{C^{o}}$$

where $F^{o}$ and $C^{o}$ represent the deformation gradient tensor and the right Cauchy–Green tensor between the initial configuration and a significantly altered in vivo state (intermediate state, e.g., the group-specific systolic blood pressure), respectively [46]. These stiffness values can be interpreted as the local slope of the stress–stretch curve at a specific intermediate, deformed configuration [48].

Finally, structural stiffness was measured through aortic distensibility, D, which relies on the pulse pressure of the groups (Eq. (1)), and the systolic and diastolic inner diameters of the vessel, denoted as $d_{i,\text{sys}}$ and $d_{i,\text{dias}}$, respectively

(13) $$D=\frac{d_{i,sys}-d_{i,dias}}{d_{i,sys}(\Delta P)}$$

### 2.6 Statistics.

Unless otherwise indicated, the data are presented as the mean value along with the standard error of the mean (SEM). We checked the normality of the data using the Shapiro–Wilk normality test. If the data did not follow a normal distribution, we evaluated statistical significance using the Mann–Whitney test, which compares the ranking of data between groups. For all other measurements, including thickness, luminal diameter, stretch, stress, stiffness, and elastic energy we used an unpaired Student’s t-test with two-tailed distributions. Any differences in the data were considered statistically significant when the *P* value ≤ 0.05.

## 3 Results

**Fig. 3:**
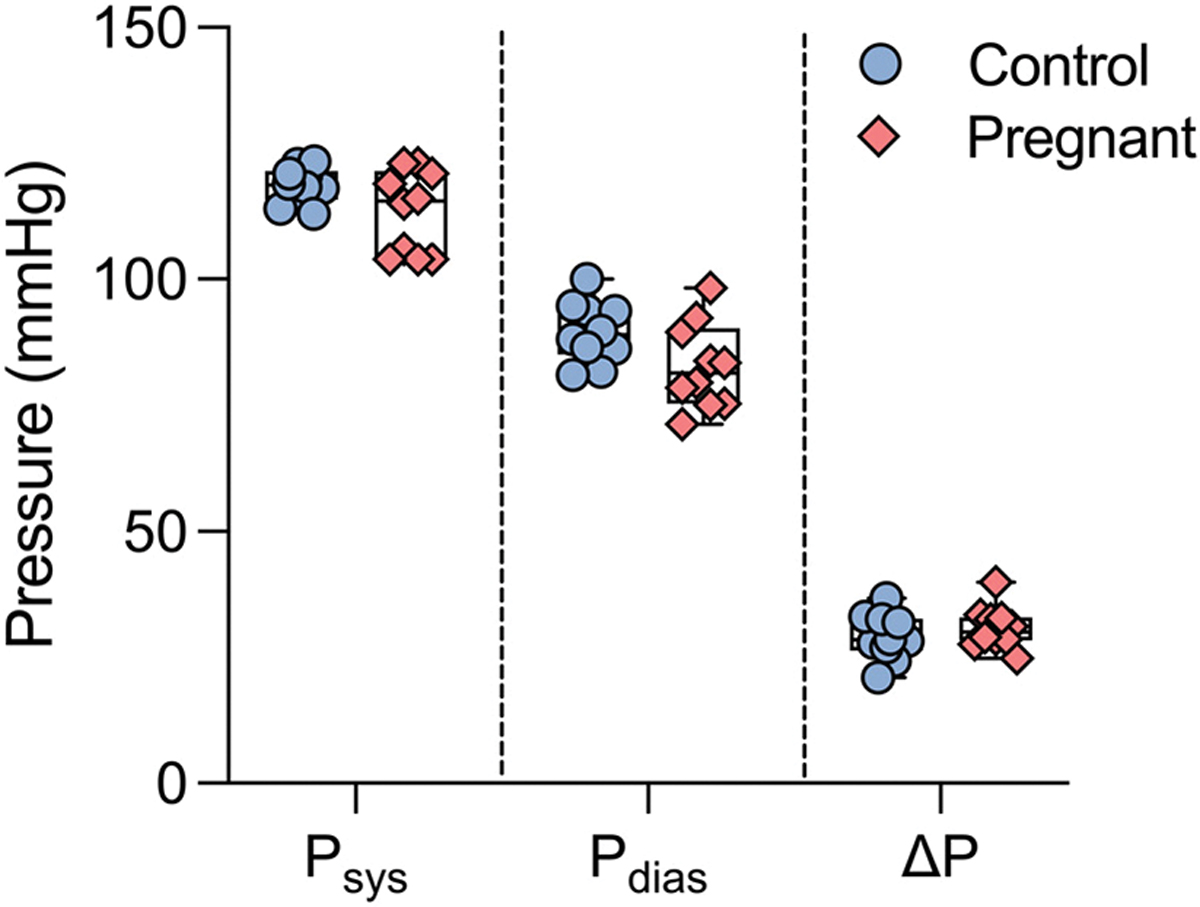
Systolic $\left(P_{\text{sys}}\right)$, diastolic $(P_{\text{dias}}$), and pulse pressure (ΔP) measurements of late-gestation pregnant (pink diamonds, n=10) and age-matched nulligravida control (blue circles, n=10) mice. Blood pressure measurements were comparable between groups. Statistical significance were determined using the Student’s t-test.

Peripheral blood pressure measurements exhibited no significant differences between the two groups (Fig. 3). The systolic blood pressure was 119 ± 1 mmHg in the control group and 114 ± 3 mmHg in the pregnant group. The diastolic blood pressure was 90 ± 2 mmHg and 83 ± 3 mmHg for the control and pregnant groups, respectively. The mean arterial blood pressure was recorded as 99 ± 2 and 93 ± 3 mmHg in the control and pregnant groups, respectively. In all pressure comparisons between control and pregnant group, *P* value was greater than 0.05.

**Fig. 4:**
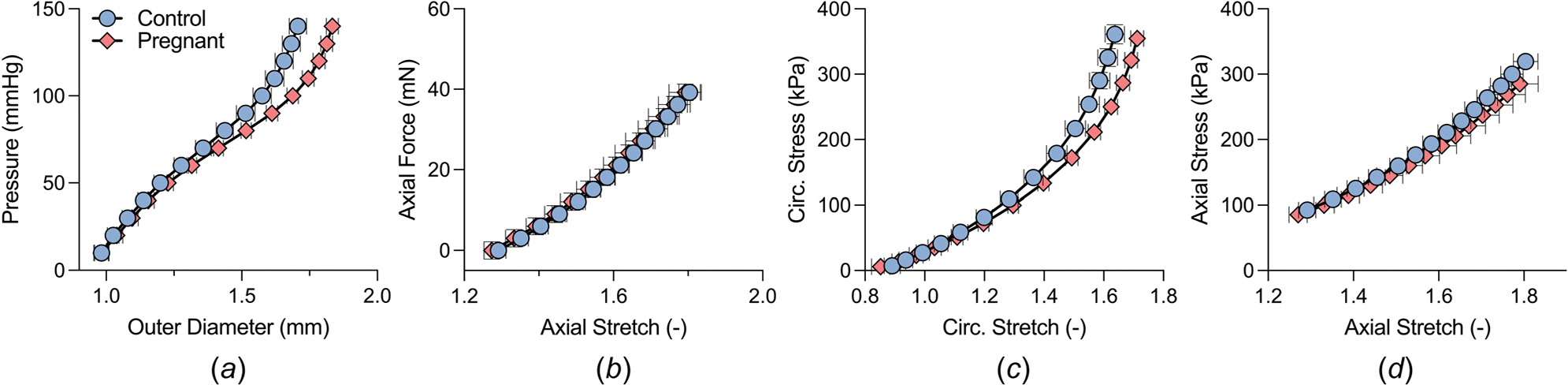
Average mechanical response of ATA tissue from late-gestation (pink diamonds, n=10) and age-matched control (blue circles, n=10) mice. (*a*) Passive pressure versus outer diameter response at group-specific in vivo axial stretch. (*b*) Axial force–stretch response at 100 mmHg transmural pressure. (*c*) Circumferential (Circ.) stress–stretch response obtained from inflation tests at group-specific in vivo axial stretch. (*d*) Axial stress–stretch response obtained from extension tests at 100 mmHg transmural pressure. The pregnant group exhibited a rightward shift from the control group in the pressure-diameter and circumferential stress–stretch responses. In contrast, the axial responses were similar for both groups. The error bars represent the standard error of the mean (SEM) based on individual data collected from tissue samples.

The mechanical responses of both control and late-gestation tissues are summarized by the best-fit material parameters listed in Table 1. These best-fit constitutive parameters serve as indicators for the typical mechanical behavior of ATA tissues across their entire thickness. Pressure–diameter curves of the ATA tissue showed that higher diameters were reached in the pregnant group at the same pressure levels (Fig. 4(a)). Such a response suggests that the pregnant aorta is more distensible, exhibiting a larger range of pressure-induced diameter change. The axial extensibility of the tissue was similar between groups (Fig. 4(b)), suggesting no significant force-induced length changes between groups. The curve representing the circumferential stress versus circumferential stretch for the pregnant group was to shifted the right of the curve representing the control group (Fig. 4(c)), suggesting global material softening of the tissue. This difference indicates that the ATA in the pregnant group experiences a larger circumferential stretch (outward expansion) than that of the control group for any given applied stress. However, the axial stress–stretch behavior (Fig. 4(d)) of the tissue showed an overlapping axial material response between control and pregnant samples, suggesting a similar axial behavior between the two groups.

**Fig. 5:**
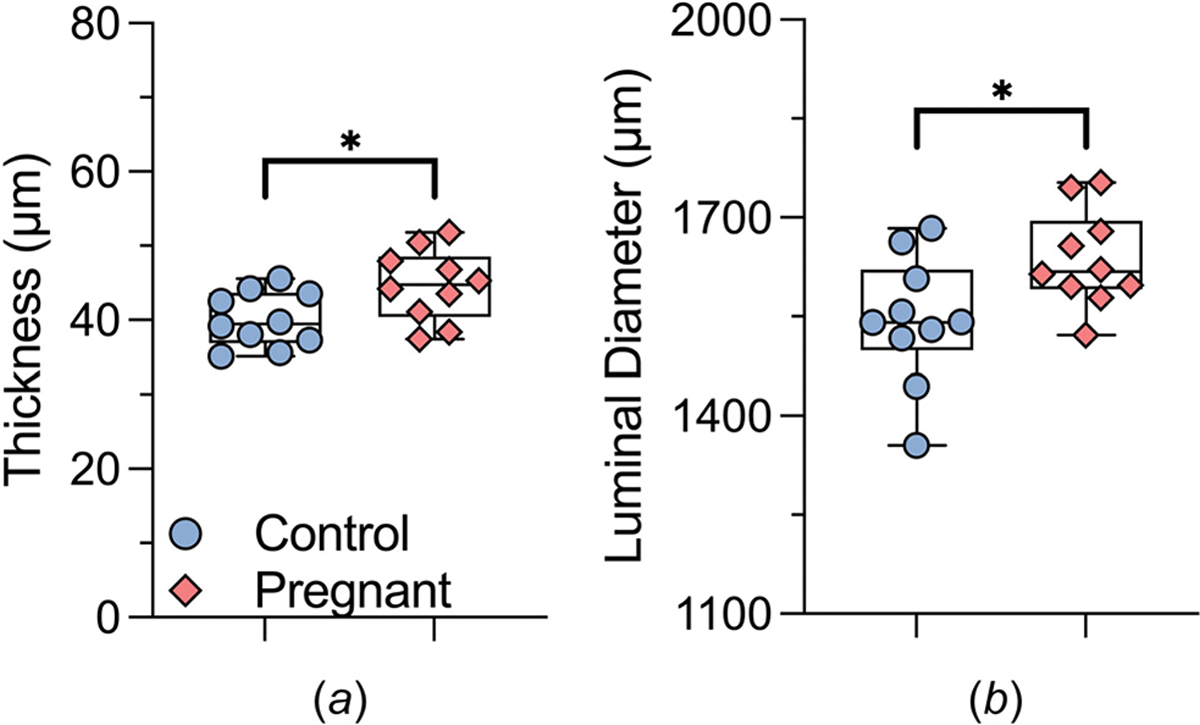
Geometric parameters of the ATA in late-gestation pregnant (pink diamonds, n=10) and age-matched control (blue circles, n=10) mice. These measurements were obtained at loaded, group-specific systolic blood pressure levels. Importantly, in alignment with our earlier finding on the descending segment of the thoracic aorta [29], an elevation in cardiac output while keeping blood pressure constant (as illustrated in Fig. 3) led to a marked outward expansion of the ATA. This expansion was marked by an augmented thickness and an enlargement in the luminal diameter. Statistically significant differences of *P* ≤ 0.05 are marked by asterisks (*).

**Fig. 6:**
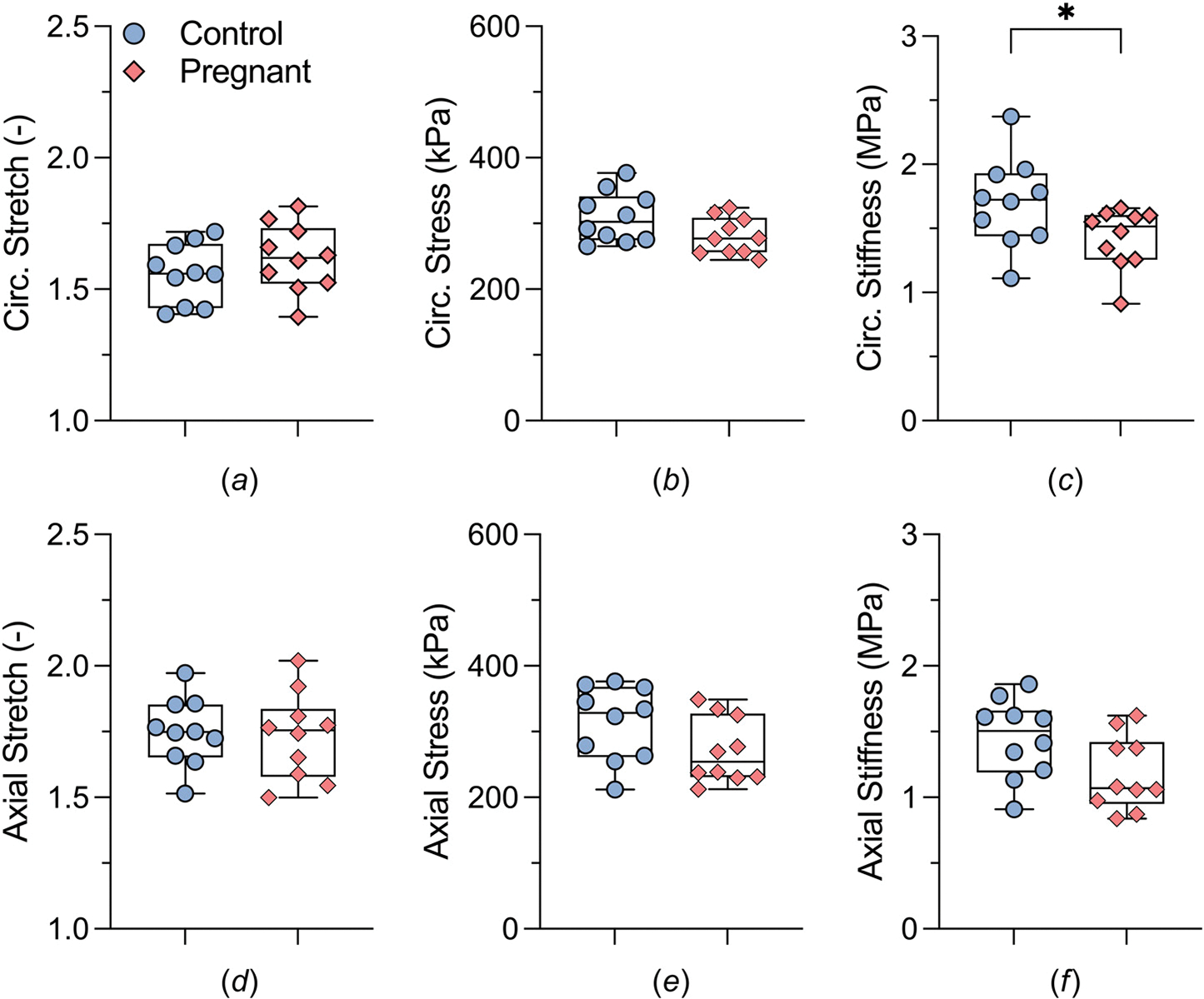
Comparative analysis of structural and material metrics in the ATA between late-gestation pregnant (represented by pink diamonds, n=10) and control (represented by blue circles, n=10) mice. Key parameters, calculated at group-specific systolic blood pressure, include stretch in both (*a*) circumferential and (*d*) axial directions, tensile stress in (*b*) the circumferential and (*e*) axial directions, and stiffness in (*c*) the circumferential and (*f*) axial directions. Statistically significant differences (i.e., *P* ≤ 0.05.) are marked with asterisks (*).

Figures 5 and 6 highlight further geometric (Figs. 5, 6(a), and 6(d)) and material (Figs. 6(b), 6(c), 6(e), and 6(f)) differences at group-specific systolic blood pressure. In particular, the pregnant tissue displayed an increase in loaded wall thickness (Fig. 5(a)), measuring 45 ± 2 *μ*m, compared to a thickness value of 40 ± 1 *μ*m in the control group. Additionally, the luminal diameter (Fig. 5(b)) in the pregnant samples expanded to 1636 ± 23 *μ*m while the control group maintained a diameter of 1544 ± 31 *μ*m. However, no significant differences were observed between the stretch values in the circumferential (1.56 ± 0.04 and 1.62 ± 0.04, for control and late-gestation samples, respectively, *P* > 0.05) or axial (1.75 ± 0.04 and 1.73 ± 0.05 for control and late-gestation samples, respectively, *P* > 0.05) directions. The increase in wall thickness (Fig. 5(a)) counterbalanced the increase in luminal diameter (Fig. 5(b)) leading to a trend toward decreased circumferential wall stress (Fig. 6(b), Eq. (3)) in the pregnant group (from 310 ± 12 to 280 ± 9 kPa, *P* = 0.07). The same was observed for the axial wall stress (Fig. 6(e)), trending to decrease from 313 ± 18 to 271 ± 16 kPa for the control and pregnant group, respectively; however, the values were not significantly different (*P* = 0.09).

**Fig. 7:**
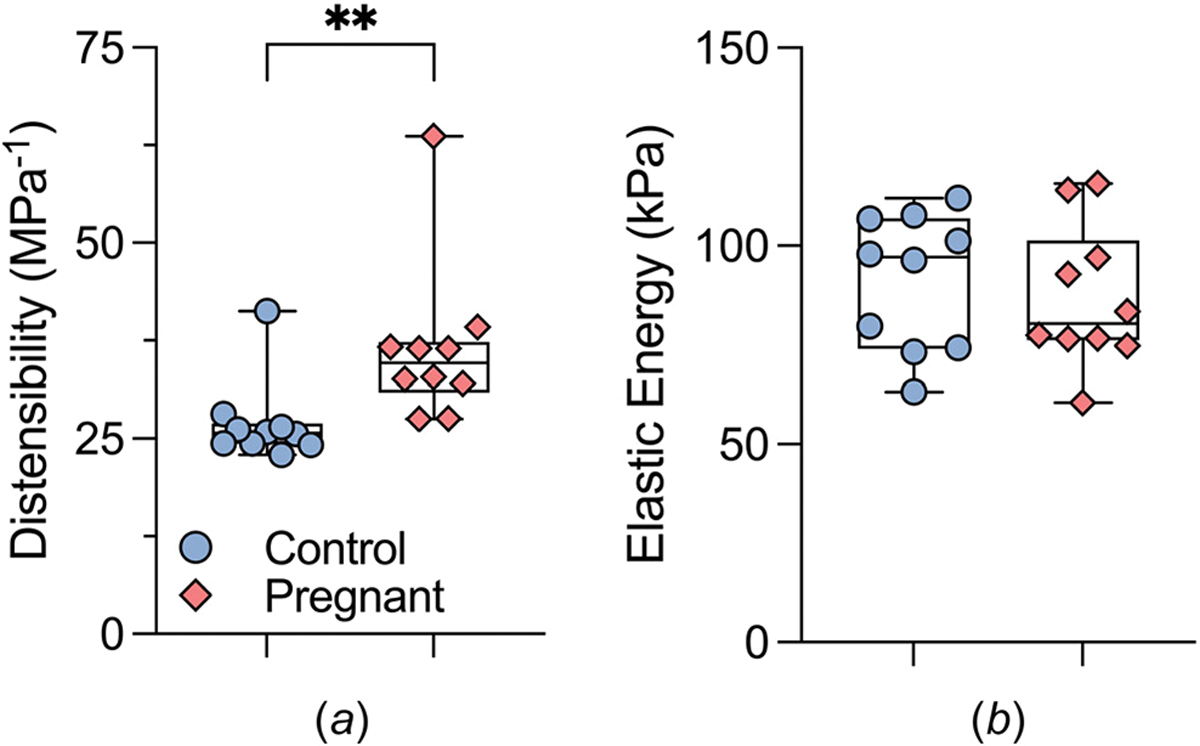
Metrics of vascular function for the ATA of control (blue circles, n=10) and late-gestation (pink diamond, n=10) wild-type mice. (*a*) Aortic distensibility serves as an indicator of structural stiffness and is calculated by using alterations in diameter and pressure that occur between systole and diastole. (*b*) Stored elastic energy density measures the capacity of the aortic wall to act as a pressure reservoir during the cardiac cycle and maintain continuous perfusion of peripheral organs and tissues. The two asterisks (**) represent a statistically significant difference of *P* ≤ 0.01.

Intrinsic stiffness in the circumferential direction (Fig. 6(c)) decreased significantly from 1.70 ± 0.11 MPa in the control group to 1.42 ± 0.07 MPa in the pregnant group (*P* = 0.05). The same trend was observed in the axial direction (Fig. 6(f)) with average stiffness values of 1.44 ± 0.10 MPa in the control group and 1.18 ± 0.09 MPa in the pregnant group; however, no significance was found between these values (*P* = 0.06). Vascular function of the ATA was assessed by measuring distensibility and capacity for elastic energy storage. Pregnancy was associated with an increase in distensibility (Fig. 7(a)), an inverse metric of structural stiffness (from 27 ± 2 to 37 ± 3 MPa^−1^), following the same trend as the intrinsic material stiffness. Elastic energy density stored within the tissue under systolic loads (Fig. 7(b)), was similar between groups, with values of 91 ± 5 kPa and 87 ± 6 kPa for the control and pregnant groups, respectively (*P* > 0.05).

## 4 Discussion

With CVD being the leading cause of maternal death in the U.S. and maternal mortality rates continuing to rise over the years [3,49], it is crucial to understand how the central vasculature remodels to accommodate for hemodynamic changes. Pregnancy is characterized by dynamic changes in the maternal circulatory system. These changes include notable reductions in total peripheral resistance and substantial increases in cardiac output [7,11–13]. In addition, emerging evidence suggests that pregnancy history has a role in predicting maternal cardiovascular health [50,51]. The objective of this work was to assess the passive mechanics of the murine ATA from C57BL/6 wild-type mice in the late-gestation period and report changes in the geometry and intrinsic stiffness of the tissue. We also compared these altered parameters to our recently published work [29], which—for the first time—provided evidence of aortic remodeling in the murine DTA.

Hemodynamic measurements in humans during gestation have revealed that throughout the course of pregnancy, cardiac output and blood volume increase [7,12,13], while blood pressure remains constant [7–9]. In addition, our previous work has established that wild-type mice serve as a physiologically relevant model to study vascular remodeling during pregnancy [29]. In line with these observations, the hemodynamics data presented in this study demonstrate that there are no significant differences in peripheral blood pressure measurements between the control and late-gestation groups (Fig. 3), further advocating for the use of wild-type mice as an appropriate model of normotensive pregnancy.

Pregnancy is associated with a notable outward expansion of the ATA, (Fig. 5), an adaptation that closely resembles the changes observed in the DTA [29]. Comparative analysis between both thoracic regions (Table 2) reveals a consistent increase in thickness during late gestation. An 11% and 13% thickness increase was observed in the DTA and ATA groups, respectively, with *P* ≤ 0.05 for comparison between late-gestation and control in each study, indicating uniform outward geometric remodeling in both aortic segments. This phenomenon could be associated with altered blood flow, which is a factor known to induce vascular remodeling [52,53]. Additionally, in late-gestation pregnancy, an increase in luminal diameter was observed both in the ATA and the DTA, a finding consistent with previous reporting of the enlargement of the aortic root diameter [54,55] and the expansion of the aortic valve orifice [7,56] during pregnancy. Similarly, the observed luminal widening could be a potential mechanism to allow the flow and cardiac output to rise, without drastically changing the circumferential tensile wall stress. This phenomenon is described by the Law of Laplace, which states that circumferential wall stress is directly proportional to vessel pressure and radius, emphasizing the ability of the vessel to adapt to changes in luminal dimensions to maintain stress homeostasis.

The observed geometric changes play a crucial role in maintaining the maternal tensile aortic wall stress both in the circumferential direction and in the axial direction within its optimal range. In other words, despite the higher cardiac output during pregnancy, the tensile wall stress did not increase, as indicated by the geometric changes observed and the Law of Laplace. In fact, the remodeling of the ATA during pregnancy even leads to a *nonsignificant trend* of decreasing circumferential and axial wall stresses (*P* = 0.07 for the comparison of the circumferential stress, and *P* = 0.09 for the comparison of the axial stress), as illustrated in Figs. 6(b) and 6(e). These findings, although similar, are less pronounced than those observed in the DTA [29] (Table 2), where circumferential and axial wall stresses significantly decreased (*P* ≤ 0.05). It is well established that increased wall stress serves as an indicator of cardiovascular disease risk or related cardiovascular events [57]. Therefore, in normotensive pregnancies, maintaining tensile stress at or below normal values might be a mechanism to maintain homeostasis and prevent significant increases in wall stress when drastic increases in blood pressure occur following gestation (i.e., during labor [58] or the postpartum period). Similarly, it has been previously suggested that central arteries reduce their axial stretch, consequently lowering their biaxial stress, as a mechanism to compensate for increased hemodynamic loads [59]. This compensatory mechanism has been observed in different regions of the aorta, which is remarkable given the large range of axial stretch values normally experienced by the different aortic segments—from 1.75 ± 0.04 in our control ATA (Table 2) to 1.54 ± 0.04 in our control DTA. In our work, the axial stretch did not change significantly between pregnant and control groups. However, the wall tensile stresses in the circumferential direction decreased, though only significantly so in the DTA but not in the ATA in the pregnant groups.

Similarly, circumferential wall stretch (Fig. 6(a)) remained comparable between both groups, suggesting that the pregnant ATA maintained consistent elasticity despite the observed decrease in circumferential intrinsic tissue stiffness (Fig. 6(c)). However, in contrast to these findings in the ATA, it is noteworthy that in the DTA, circumferential wall stretch increases (Table 2) during late-gestation. This difference between both of these anatomical directions might be due to proximal versus distal differences. Specifically, while the ATA is generally more elastic due to its proximity to the heart and exposure to higher pressures, the DTA may have a higher proportion of collagen fibers and less elasticity [60]. This difference in wall composition could result in a greater circumferential stretch of the DTA when compared to the ATA.

Based on our findings, the pregnant ATA wall and its tissues became more deformable in the circumferential direction (Figs. 4(a) and 4(c)). Notably, the late-gestation group displayed a statistically significant decrease in the circumferential intrinsic tissue stiffness when compared to the age-matched control group. Similarly, the structural stiffness of the pregnant ATA, inversely related to aortic distensibility (Fig. 7(a)), also exhibited a reduction as compared to that of the control group. These observations are consistent with prior findings in the DTA [29] (Table 2) and align with clinical studies that have reported a decrease in aortic structural stiffness, typically measured through pulse wave velocity [61,62]. Conversely, the axial response of the ATA between both the control and pregnant group remained similar, as indicated by the overlapping axial force–stretch and stress–stretch curves seen in Figs. 4(b) and 4(d). In contrast, in our recent study of the pregnancy-induced changes in murine DTA [29], we observed tissue softening in the axial direction and a significant reduction in the stiffness (Table 2). One could explain such differences between the ATA and DTA by examining their anchoring to the adjacent tissues. The ATA experiences substantial stretching during each cardiac cycle, in part, because of the continuous axial movement of the heart. The DTA is, however, tightly tethered to the thoracic spine and therefore does not change its length within every cardiac cycle [59]. One could therefore speculate that the ATA may be better predisposed or equipped to accommodate changes in hemodynamic loads during pregnancy while the DTA requires adjustment of tissue properties to maintain physiological function.

The primary function of large arteries lies in their ability to serve as energy reservoirs, storing elastic energy during systole and using that energy to maintain blood circulation during diastole [63,64]. Loss or decrease of this energy is an indicator of vascular dysfunction and has been connected to aortic disease [24,39,65]. Our data indicate that pregnancy does not significantly alter the capacity for elastic energy storage of the tissue, suggesting that vascular function both in the ATA (Fig. 7(b)) and in the DTA (Table 2) is preserved during pregnancy. In other words, the lack of pregnancy-induced change in stored elastic energy indicates the ability of the aorta to serve as a blood reservoir, supporting the workload of the heart and preventing damage to the microvasculature [64,66].

In considering the limitations of our study, it is essential to note that our experimental methods primarily capture the passive response of the tissue, excluding the vasodilation or vasoconstriction effects. While distinguishing between growth/remodeling events and acute vasodilation remains a challenge in our current methodology, future investigations, incorporating assessments of active properties as suggested by Murtada et al. [67], could provide valuable insights into differences in smooth muscle activities between pregnant and nulligravida tissues. Additionally, while we take thorough steps to minimize permanent deformation of our tissue during the biaxial mechanical testing, it is essential to acknowledge that the tissue still experiences residual stresses in the traction-free configuration following ring extraction [68]. Despite efforts to reduce these stresses through radial and axial cuts, complete removal remains challenging due to microstructural deposition stretches [69]. This limitation underscores the challenges associated with capturing the true unloaded configuration in biological materials. Finally, while our experimental setup comprehensively replicates passive physiological loading conditions in the ATA through progressive circumferential stretch and axial lengthening under fixed luminal pressure, it is important to note that torsion loading was not included in our in vitro biaxial testing protocol.

In summary, the present results provide a detailed description of how the passive biaxial mechanical responses of the ascending portion of the thoracic aorta are different during a normotensive, late-gestation pregnancy as compared to the responses of the tissues obtained from age-matched nulligravida control mice. The murine ATA followed a similar remodeling process as previously observed in the DTA [29]. Despite no significant changes in pulse pressure during pregnancy, the ATA experienced outward remodeling, by becoming thicker and increasing its luminal diameter. These geometric changes supported a trend toward lowering tensile wall stress, coupled with decreased values of the circumferential structural stiffness of the aortic wall. The observed pregnancy-induced vascular remodeling maintained the tissue capacity for elastic energy storage, thereby indicating that vascular function in normotensive pregnant mice is preserved, despite increases in hemodynamic loads.

## Figures and Tables

**Table 1:** Best-fit material parameters calculated using average experimental data to characterize the group-specific passive properties of ATA tissues. These properties include the contribution of elastic fibers, collagen, and smooth muscle cells (SMCs) both in nulligravida control mice and in late-gestation pregnant mice.

Circumferential									
	Elastic fiber	Axial collagen		Collagen + SMC		Diagonal collagen			Error
	c (kPa)	*c*_1_^1^ (kPa)	*c*_2_^1^	*c*_1_^2^ (kPa)	*c*_2_^2^	*c*_1_^3,4^ (kPa)	*c*_2_^3,4^	α_0_ (deg)	RMSE

Control	18.9	12.2	2.3 × 10^−14^	4.16	2.1 × 10^−11^	28.5	0.193	53.7	0.0668
Pregnant	22.9	7.44	2.8 × 10^−9^	3.2 × 10^−3^	1.48	25.2	0.142	52.2	0.0700

**Table 2:** Geometric and mechanical properties (mean±SEM) of the DTA (data adapted from Vargas et al. [29]) and ATA for control and late-gestation pregnant C57BL/6 mice

*n*	DTA				ATA			
	Control 9	Late-gestation 9		% Change	Control 10	Late-gestation 10		% Change

Systolic blood pressure (mmHg)^a^	118±1	113±3	ns		119±1	114±3	ns	
Diastolic blood pressure (mmHg)	89±2	82±3	ns		90±2	83±3	ns	
Distensibility (MPa^−1^)	19±1	21±1	*	16	27±3	37±3	*	35
***Unloaded dimensions***								
Outer diameter (*μ*m)	844±14	869±13	ns		1128±24	1168±32	ns	
Wall thickness (*μ*m)	89±1	107±2	*	20	108±1	124±1	*	15
Unloaded wall length (mm)	4.1±0.2	4.4±0.4	ns		1.9±0.1	2.0±0.1	ns	
***Loaded dimensions*** @***Systolic blood pressure***								
Outer diameter (*μ*m)	1253±17	1331±27	*	6	1624±30	1726±25	*	6
Wall thickness (*μ*m)	36±1	40±1	*	11	40±1	45±2	*	13
Stretch								
Circumferential	1.62±0.02	1.69±0.02	*	4	1.56±0.04	1.62±0.04	ns	
Axial	1.54±0.02	1.57±0.04	ns		1.75±0.04	1.73±0.05	ns	
Cauchy stress (kPa)								
Circumferential	260±5	234±8	*	−10	310±12	281±9	ns	
Axial	255±7	214±9	*	−16	313±18	271±16	ns	
Linearized stiffness (MPa)								
Circumferential	1.9±0.1	1.7±0.1	*	−11	1.7±0.1	1.4±0.1	*	−18
Axial	2.3±0.1	1.8±0.2	*	−22	1.4±0.1	1.2±0.1	ns	
Stored energy (kPa)	66±2	62±4	ns		91±5	87±6	ns	

a These values are not statistically significant between ATA and DTA groups. Comparing systolic blood pressure between the control ATA group and the control DTA group led to a *p*-value of 0.87. Comparison of the systolic blood pressure between the late gestation ATA and DTA groups yielded a p-value of 0.89.

Statistical significance between control and late-gestation groups for each region of the aorta is denoted by * for *p* < 0.05. “ns” stands for nonsignificant differences between control and late-gestation groups.

**Table 3:** Hemodynamic and geometric parameters of the ascending thoracic aorta in control (*n* = 10), late-gestation (*n* = 10), and hypertensive (*n* = 8) [39] groups. Outer diameter (*d_o_*) and aortic wall thickness (*h*) were calculated at loaded systolic (sys) and diastolic (dias) conditions. All data are means of measurements in each group.

Groups	P_sys_(mmHg)	*d_o_*_,sys_(*μ*m)	*h*_sys_(*μ*m)	P_dias_(mmHg)	*d_o_*_,dias_(*μ*m)	h_dias_(*μ*m)

Control	119	1624	40	90	1488	44
Pregnant	114	1726	45	83	1526	51
Hypertensive	134	1910	72	92	1823	76

## Data Availability

All relevant data related to the work presented in this article can be obtained from the corresponding authors upon reasonable request.

## Appendix

### Educational Materials: Biomechanical Significance of Aortic Distensibility.

In our recent publications [29,70–73], we have included educational components to broaden the impact of our research. In a similar effort, the “homework problem” presented below aims to help students assess their knowledge of aortic wall biomechanics after reading the research segment of the paper. This assignment is designed for an introductory undergraduate-level biomechanics course.

Arterial compliance, referring to the ability of blood vessels to expand in response to the rhythmic flow of blood, is important in blood pressure modulation [74–77]. The role of the aorta in cushioning pulsatile blood flow may be compromised when the aortic wall undergoes structural modifications, resulting in an increased stiffness. A stiffer aorta necessitates the left ventricle to exert greater effort in overcoming the downstream resistance [77], thus overloading the heart and causing detrimental issues such as cardiac hypertrophy [78]. Aortic distensibility serves as a standard measure of aortic structural stiffness (Sec. 2.5). The aortic distensibility is calculated using the diameter and the differences between systolic pressure and diastolic pressure as shown in Eq. (13).

#### Problem.

Table 3 provides the average raw data necessary to compute the inner diameter and distensibility measurements for three groups: control, pregnant, and hypertensive. Hypertensive data are from Farra et al. [39], who induced hypertension in mice by cigarette exposure. Calculate the distensibility and structural stiffness (inverse of distensibility) for the three groups and discuss how blood pressure and geometry affect the elastic behavior of the aortic wall.
